# Impact of low injected activity on data driven respiratory gating for PET/CT imaging with continuous bed motion

**DOI:** 10.1002/acm2.13619

**Published:** 2022-04-28

**Authors:** Joseph G. Meier, Radwan H. Diab, Trevor M. Connor, Osama R. Mawlawi

**Affiliations:** ^1^ Department of Imaging Physics MD Anderson Cancer Center Houston USA; ^2^ MD Anderson Cancer Center UTHealth Science Center, Houston Graduate School of Biomedical Sciences Houston Texas USA; ^3^ Department of Internal Medicine Kansas University School of Medicine Wichita Kansas USA

**Keywords:** low injected activity SUV quantification, PET/CT respiratory motion correction data driven gating

## Abstract

Data driven respiratory gating (DDG) in positron emission tomography (PET) imaging extracts respiratory waveforms from the acquired PET data obviating the need for dedicated external devices. DDG performance, however, degrades with decreasing detected number of coincidence counts. In this paper, we assess the clinical impact of reducing injected activity on a new DDG algorithm designed for PET data acquired with continuous bed motion (CBM_DDG) by evaluating CBM_DDG waveforms, tumor quantification, and physician's perception of motion blur in resultant images. Forty patients were imaged on a Siemens mCT scanner in CBM mode. Reduced injected activity was simulated by generating list mode datasets with 50% and 25% of the original data (100%). CBM_DDG waveforms were compared to that of the original data over the range between the aortic arch and the center of the right kidney using the Pearson correlation coefficient (PCC). Tumor quantification was assessed by comparing the maximum standardized uptake value (SUVmax) and peak SUV (SUVpeak) of reconstructed images from the various list mode datasets using elastic motion deblurring (EMDB) reconstruction. Perceived motion blur was assessed by three radiologists of one lesion per patient on a continuous scale from no motion blur (0) to significant motion blur (3). The mean PCC of the 50% and 25% dataset waveforms was 0.74 ± 0.18 and 0.59 ± 0.25, respectively. In comparison to the 100% datasets, the mean SUVmax increased by 2.25% (*p* = 0.11) for the 50% datasets and by 3.91% (*p* = 0.16) for the 25% datasets, while SUVpeak changes were within ±0.25%. Radiologist evaluations of motion blur showed negligible changes with average values of 0.21, 0.3, and 0.28 for the 100%, 50%, and 25% datasets. Decreased injected activities degrades the resultant CBM_DDG respiratory waveforms; however this decrease has minimal impact on quantification and perceived image motion blur.

## 1 INTRODUCTION

Clinical positron emission tomography (PET) imaging of the abdominothoracic regions typically lasts between 2 and 3 min per bed position and can suffer from degraded image quality due to respiratory motion blur during data acquisition. Respiratory motion blur can negatively impact patient management due to underestimated activity concentration, decreased lesion detectability, and overestimated lesion volume measurements.^1^, ^2^, ^3^

Multiple methods exist to correct for respiratory motion blur in PET imaging. The majority of these methods require the acquisition of a respiratory waveform.^1^, ^4^, ^5^, ^6^, ^7^, ^8^, ^9^, ^10^, ^11^, ^12^, ^13^, ^14^, ^15^, ^16^ External devices have been primarily used to acquire the waveforms. One such system is the AZ‐733V respiratory gating system (Anzai Medical, Tokyo, Japan) in which an elastic belt with a load cell is placed around the chest or abdomen of the patient and measures a signal related to the belt tension as the patient breathes in and out. External devices such as the Anzai system, however, have technical challenges in that they require additional time for setup and troubleshooting and are prone to user setup error and hardware failure.^17^ In addition, several other studies have shown that the chest or abdominal wall motion captured by external devices does not always represent the motion of the internal anatomy,^18^, ^19^, ^20^ rendering such devices questionable regarding their ability to consistently correct for respiratory motion blur.

Data driven gating (DDG), which relies on determining the respiratory waveform entirely from the acquired PET data itself, has been introduced as an alternative to external devices to record the respiratory waveform.^21^ DDG is based on the knowledge that organs or lesions, which exhibit radiotracer uptake and are under the influence of respiratory motion, will cause periodic changes in the acquired PET data that can be detected and used to determine a respiratory waveform.^22^ DDG eliminates the need for extra time to set up external devices and does not depend on the technique or skill of the technologist attaching hardware to the patient. In addition, the DDG signal is determined from the motions of the internal anatomy in areas with radiotracer uptake, as opposed to the small motions of external surfaces.

Many different types of DDG techniques exist and have been in development over the past decade.^22^, ^23^, ^24^, ^25^, ^26^, ^27^, ^28^, ^29^, ^30^ All of these DDG methodologies have been developed exclusively for PET data acquired in Step and Shoot mode. However, no DDG method has been developed for PET data acquired in continuous bed motion (CBM)^31^ until very recently by Schleyer et al.^32^ In CBM acquisitions, available only on Siemens PET Systems with the Flow Motion option (Siemens Medical Solutions USA, Inc),^33^ the patient is continuously translated through the scanner, while in comparison to Step and Shoot, the patient is imaged in multiple stationary and overlapping bed positions.^34^ The approach by Schleyer et al.,^32^ which uses a spectral analysis method,^28^ was originally developed for Step and Shoot, but later adapted to work with CBM data^32^ in order to account for the combination of anatomical motion due to both respiratory motion and continuous bed motion.

Recently, Büther et al. published the first clinical study, which characterized the impact of this CBM DDG (CBM_DDG) algorithm on tumor quantification, waveform characteristics, and visual assessment of motion blur.^35^ However, their investigation did not assess the impact of decreased administered activity on the ability of the CBM_DDG algorithm to extract the respiratory waveform, a situation that has been shown to degrade the performance of DDG algorithms.^30^, ^36^ Such an investigation is also important due to the growing interest in performing PET studies with low injected activity (low count rate) with special focus on the pediatric population,^37^, ^38^ dual and multi‐time point imaging,^39^ as well as performing Y‐90 radioembolization postadministration dosimetry, which has relatively low count rates.^40^

In this work, we evaluate the impact of decreased injected activity or scan duration on this novel CBM_DDG algorithm in patient studies. The evaluation is based on comparing waveforms, quantitative tumor measurements, and physician evaluation of images processed with the CBM_DDG algorithm. To our knowledge, this investigation is the first to perform a comprehensive clinical evaluation while using the CBM_DDG algorithm with simulated decreased injected activity. We have previously published the initial results of this investigation in an abstract form.^41^, ^42^

## 2 METHODS

### 2.1 Continuous bed motion data driven gating algorithm

**FIGURE 1:**
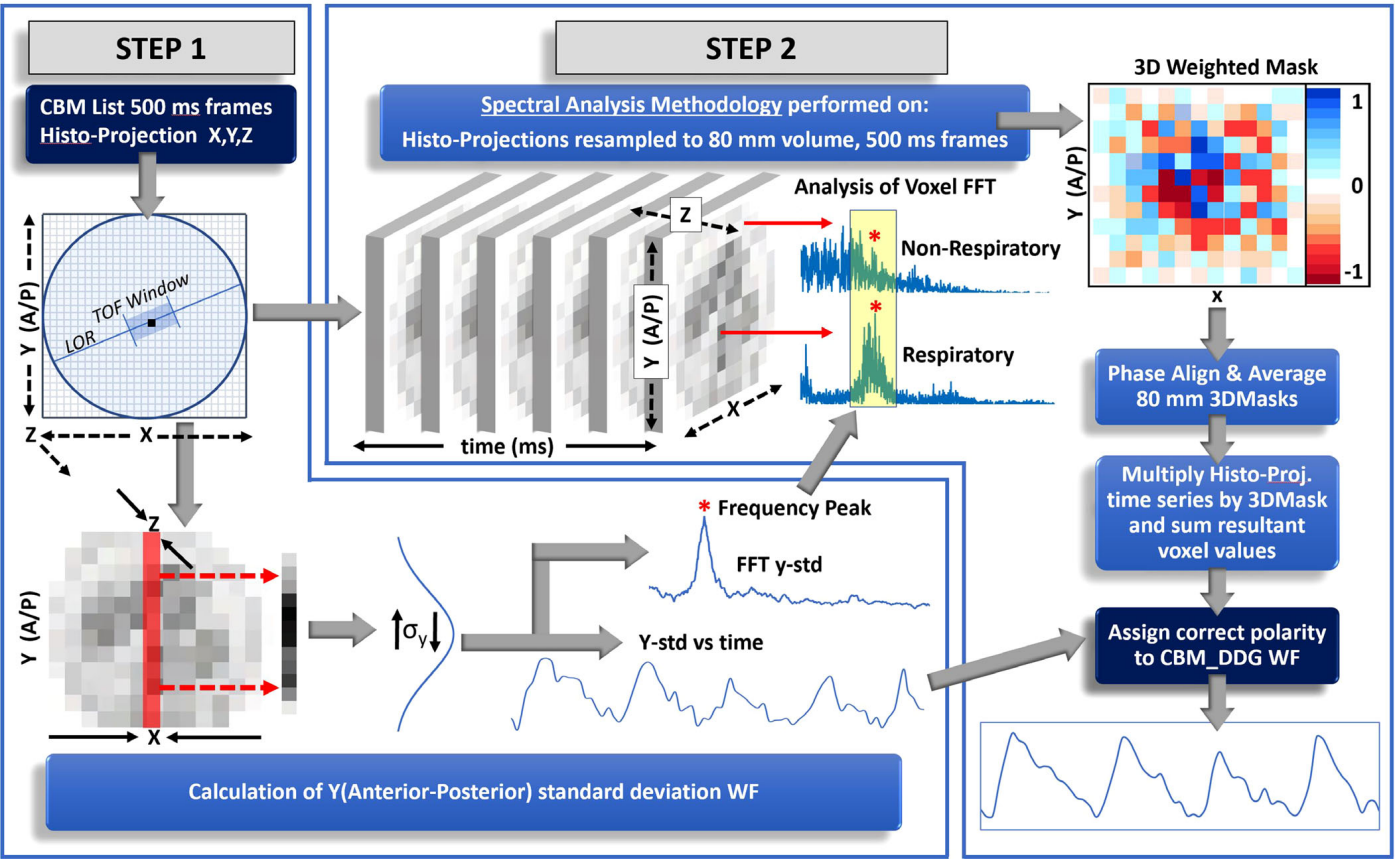
Overview of the continuous bed motion_data driven respiratory gating (CBM_DDG) workflow. WF = waveform. For the histo‐projections, the transaxial dimensions are represented by the x(medio‐lateral) and y(anterior‐posterior) dimensions, while the axial dimension is the z(superior‐inferior) dimension

The CBM_DDG algorithm has been previously introduced.^32^ Here, we briefly describe its general framework. The algorithm is composed of a two‐step process: Step 1 starts by dynamically framing the list mode dataset into a time series of 500 ms frames. Figure 1 shows that for each frame a histo‐projection volume is created by placing each recorded coincidence event at the center of its respective time of flight window. Each event is also placed along its respective line of response. Each one of these histo‐projection volumes is then projected onto the y‐axis, and the standard deviation for each projected 500 ms frame is calculated. At inspiration, the standard deviation of the collapsed frames will be highest, and as the patients breathe, the standard deviation along the anterior posterior direction will vary periodically. The Fast Fourier Transform of this y‐standard deviation respiratory waveform is used to estimate the global respiratory frequency, which is subsequently utilized to define the frequency peak in step 2 of the algorithm.

In step 2, the 500‐ms histo‐projection volumes created from the entire axial extent of the CBM acquisition are then resampled into 80‐mm axial range volumes, which are overlapped by 70 mm. From each 80 mm volume, the spectral analysis method^28^ creates a 3D weighted mask to include only the signal from regions influenced by respiratory motion. All masks are then averaged into one final 3D weighted mask, which encompasses the entire axial scan range while correcting for phase offsets between masks. The 3D weighted mask is then multiplied by the consecutive 500‐ms histo‐projection volumes, and the resultant voxels are summed to generate one time point value of the DDG waveform. In the final step, the correct signal polarity is assigned to the CBM_DDG waveform as described in further detail by Schleyer et al.^32^

### 2.2 Patients

Forty patients (19 males and 21 females mean age: 63.7 ± 13.3; mean body mass index (BMI): 28.1 ± 6.8) having at least one lung or liver tumor less than 3 cm in diameter and located in regions impacted by respiratory motion were recruited for this study (MD Anderson Cancer Center Internal Review Board 2015‐0989). Informed consent was obtained prior to imaging. Patients were instructed to fast for 6 h before injection of 320.7 ± 54.4 MBq ^18^F‐FDG. The injection to scan delay time was 69.8 ± 9.5 min.

### 2.3 PET scan and image reconstruction

All patient scans were acquired on a four‐ring Siemens Biograph mCT Flow system^33^ (Siemens Medical Solutions USA, Inc). A computed tomography (CT) topogram of 120 kVp and 20 mA, followed by a 3D helical CT scan using CARE kV(120 reference kV), tube current modulation with Care Dose4D(90 reference mAs), pitch of 1.4:1, 16 × 1.2 mm beam width, and gantry rotation time of 0.5 s, was acquired according to our clinical protocol. The 3D helical CT was acquired with free‐breathing, and the PET data were acquired with CBM using a table speed of 0.8–1 mm/s depending on the patient BMI. For the lung/liver region ‐ areas that are affected by breathing motion ‐ a table speed of 0.5 mm/s was prescribed. The Anzai AZ‐733V respiratory gating system (Anzai Medical Co.) system was used to acquire all external device respiratory waveforms, while the CBM_DDG algorithm was used to derive the respiratory waveform from the PET list data.

The impact of lower injected activity on the CBM_DDG algorithm was simulated by randomly removing events from the original list mode‐dataset resulting in data sets, which retained 50% and 25% of the original list‐data. For all patients and their respective 100%, 50%, and 25% list mode datasets, two respiratory motion correction reconstructions were performed using the elastic motion deblurring (EMDB)^5^ algorithm which is known commercially as Siemens OncoFreeze. One of the EMDB recons used the Anzai waveform (ANZ_EMDB), and the other used the DDG waveform (DDG_EMDB). In both cases, the EMDB algorithm used a 35% duty cycle to reconstruct the HDChest reference images. For both reconstructions, the following parameters were used: two iterations, 21 subsets, time‐of‐flight information, point spread function correction, 200 × 200 matrix, 4.07 mm × 4.07 mm × 2.03 mm voxel size, and 5 mm full‐width at half‐maximum isotropic Gaussian postfiltration. The ANZ_EMDB reconstruction was used only for the subjective radiologist evaluation as described in the next section. Creation of the list mode datasets with fewer coincidence events, calculation of the CBM_DDG waveforms, and image reconstruction were all performed with the Siemens research e7 tools software.

### 2.4 Patient evaluations

Two objective analyses were used to evaluate the CBM_DDG algorithm: (1) waveform comparisons and (2) image quantification. Additionally, we performed a subjective analysis of image quality by detecting the presence of motion blur in the resultant reconstructed images as assessed by radiologists.

For the waveform comparison, we calculated the Pearson correlation coefficient (PCC) to compare the 100% to the 50%, the 100% to the 25 %, as well as the 50% to the 25% CBM_DDG respiratory waveforms for each patient, and the results were averaged and compared. The waveforms were compared for the time segment between the aortic arch and the center of the right kidney because these anatomical locations are impacted the most by respiratory motion.

To assess the impact of varying injected activity on tumor quantification when using the DDG_EMDB motion correction algorithm, the maximum standardized uptake value (SUVmax) and peak SUV (SUVpeak) were measured, and the percent change of the 50% and 25% DDG_EMDB reconstructions was calculated with respect to the 100% DDG_EMBD reconstruction. At the time of the examination, six patients’ tumors had resolved with respect to the baseline scans or did not have discernable uptake and so measurements of FDG foci (renal medullae (4), spleen (1), and gastrointestinal (1)) impacted by respiratory motion were made. Only one measurement per patient was made to avoid bias from patients with multiple tumors.

To assess the impact that injected activity has on the ability of DDG_EMDB to visually reduce motion blur, a subjective assessment was made by three radiologists experienced in PET/CT interpretation. In this evaluation, the ANZ_EMDB and the DDG_EMDB were used. The reconstructed images were separated into three independent groups containing only the: (1) 25% list‐mode images, (2) 50% list‐mode images, or (3) the 100% list‐mode images. Only one of the groups was assessed at a time. For each group, only one patient was assessed at a time and with a custom workflow, coronal images of both the ANZ_EMDB and DDG_EMDB reconstructions were displayed side by side in a randomized left to right order. DDG_EMDB images of different list‐mode data fractions were not directly compared to one another as the stark differences in image noise could have biased the interpretation. For each patient, the radiologists were asked to assess if there was any difference in motion blur for a specific lesion when comparing the ANZ_EMDB and DDG_EMDB reconstructions. If there was no difference, both image series were assigned a score of zero. If one image series had more blur, then it was scored on a continuous scale (no motion blur (0), slightly more motion blur (1), moderately more motion blur (2), and significantly more motion blur (3)). The image series with less blur was scored with a zero. In this regard, a mean score of zero implies that the DDG_EMDB images are visually similar in quality to those of the ANZ_EMDB. The intrareader reliability of each reader was assessed by repeating 10 randomly selected patient studies. The order of patient presentation was randomized for all three groups independently. For each group, the mean motion blur score was calculated for the DDG_EMDB reconstructions, and the percent change of the 50% and 25% DDG_EMDB motion blur scores was calculated with respect to the 100% DDG_EMBD motion blur score.

### 2.5 Statistical analysis

All statistical analyses were performed using the R computing language (version 3.6.1). Wilcoxon signed ranked tests with Benjamini–Hochberg corrections were performed to test the statistical significance of differences between the CBM_DDG results. A two‐way random effects, absolute agreement, multiple raters intraclass correlation coefficient was used to assess interreader reliability regarding the respiratory motion blur scores, whereas a two‐way mixed effects, absolute agreement, multiple raters intraclass correlation coefficient was used to assess intrareader reliability regarding these scores.^43^ The values were interpreted as ICC < 0.5 (poor), 0.5 < ICC < 0.75 (moderate), 0.75 < ICC < 0.9 (good), and 0.9 < ICC < 1.0(excellent).^43^

## 3 RESULTS

### 3.1 Patient waveform comparison and image quantification

**FIGURE 2:**
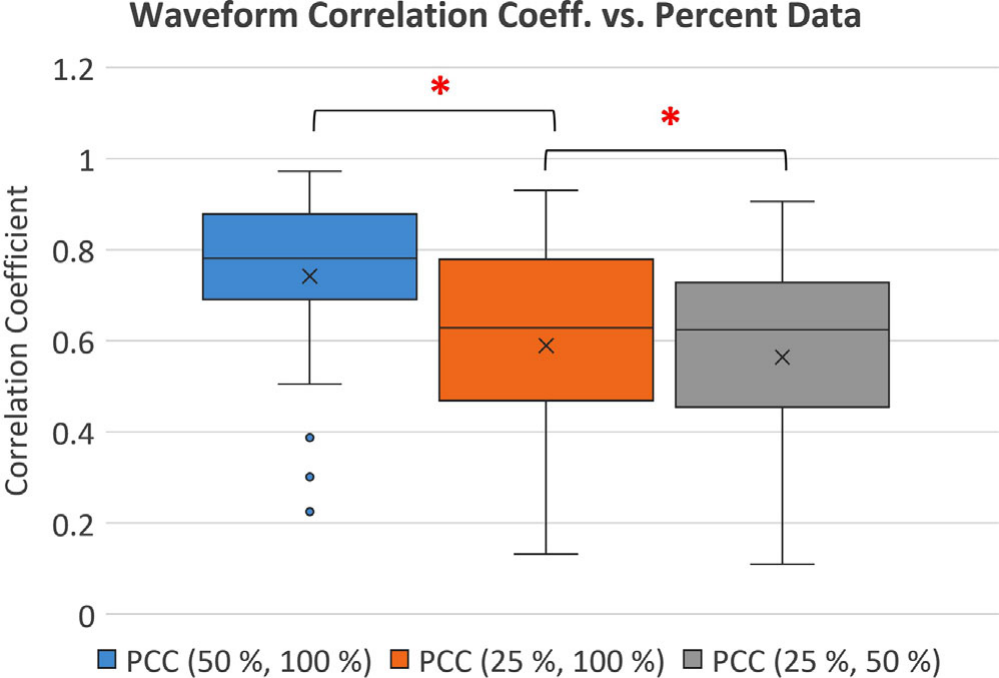
Boxplots of the Pearson correlation coefficients of the waveform from the 50% and 25% datasets continuous bed motion_data driven respiratory gating (CBM_DDG) in comparison with the 100% CBM_DDG dataset. The comparison was made on the time segment of the waveform acquired over the aortic arch to the center of the right kidney. An x indicates the mean value, and a star indicates *p* < 0.002

Comparison of the 50% CBM_DDG and the 25% CBM_DDG waveforms to the corresponding 100% CBM_DDG as well as comparison of the 50% CBM_DDG to the 25% CBM_DDG waveforms showed that the mean +/− standard deviation of the PCC decreased as the percentage of list mode data used decreased. The mean (± standard deviation) of the PCCs was 0.74 ± 0.18 between the 50% CMB_DDG and the 100% CBM_DDG waveforms, 0.59 ± 0.25 between the 25% CMB_DDG and the 100% CBM_DDG waveforms, and 0.56 ± 0.24 between the 50% CMB_DDG and the 25% CBM_DDG waveforms as seen in Figure 2. The mean PCC between the 25% and the 100% datasets decreased by −20.27 ± 30.17% (*p* < 0.000) in comparison to the mean PCC between the 50% and the 100 % datasets. The mean PCC between the 25% and the 50% datasets decreased by −5.08 ± 37.24% (*p* < 0.002) in comparison to the mean PCC between the 25% and the 100% dataset.

**FIGURE 3:**
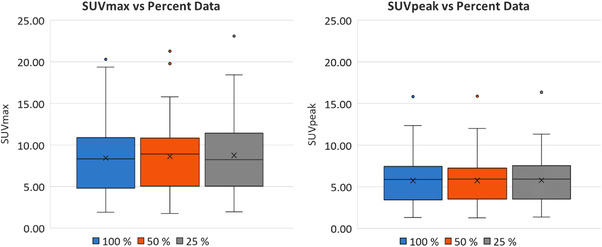
Boxplots of SUVmax and SUVpeak for the F18‐FDG foci with respect to the 100%, 50%, and 25% datasets. In comparison to the 100% dataset, the differences in means were not statistically significant for the 50% and 25% datasets, respectively. An x indicates the mean value

The results of the SUVmax and SUVpeak are shown in Figure 3. The results show that as the percent data decrease to 50% and 25%, the SUVmax increases by 2.25% (*p* = 0.11) and 3.91% (*p* = 0.16), respectively. However, this increase was not found to be statistically significant. SUVpeak results showed that there was negligible change with percent data decrease.

**FIGURE 4:**
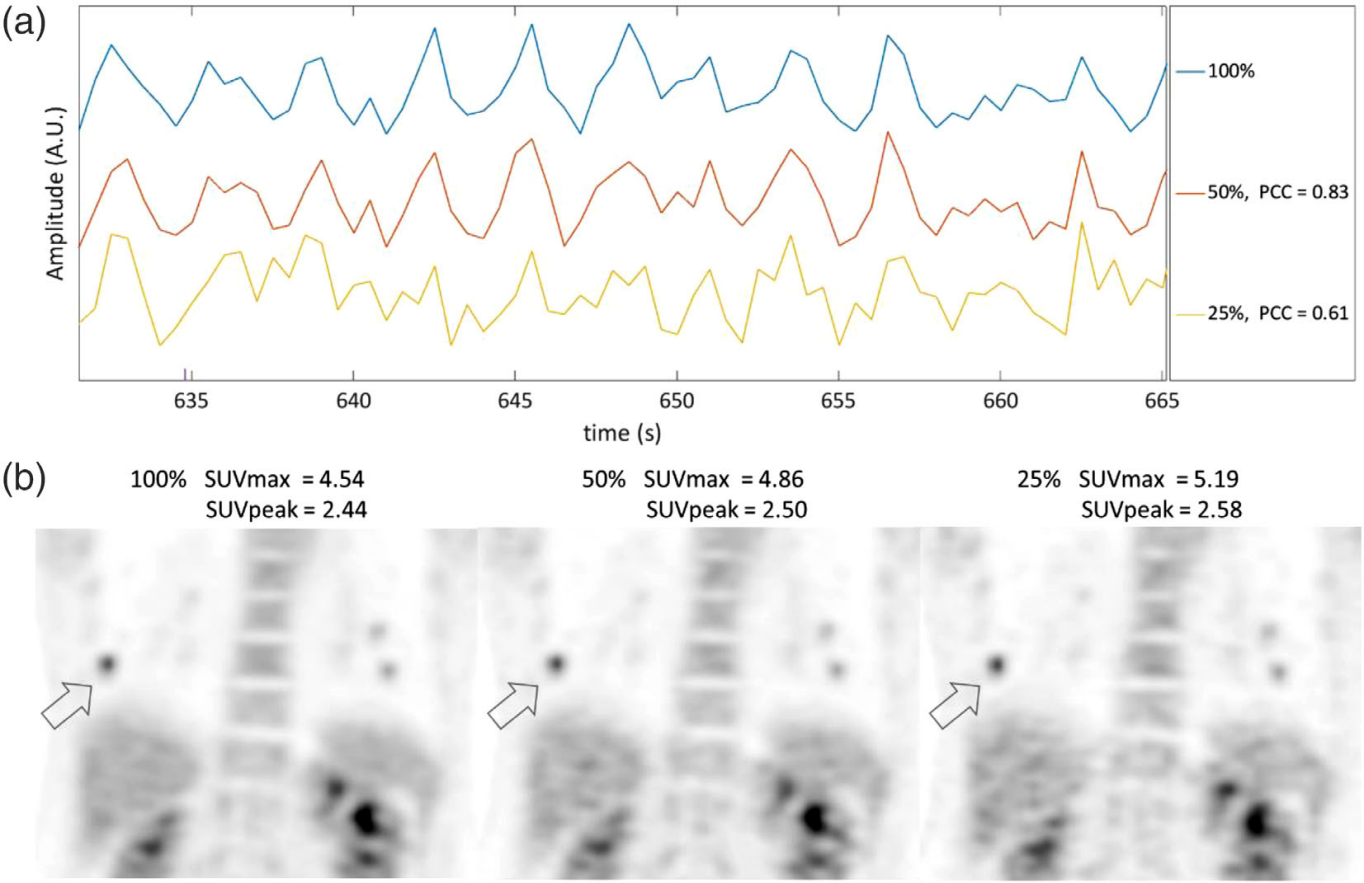
(a) Representative continuous bed motion_data driven respiratory gating (CBM_DDG) respiratory waveforms when using 100 %, 50%, and 25% of the list mode data along with respective correlation coefficients. (b) SUVmax of a lower right lung tumor is measured showing that as the amount of data used decreases SUVmax and SUVpeak increase

Figure 4 shows waveforms and images of an example patient with decreasing percent data. Figure 4a shows that the PCC decreases with decreasing percent data, while Figure 4b shows that SUVmax increases with decreasing data, while SUVpeak has smaller increases.

### 3.2 Physician visual assessment

Table 1 shows the mean, standard deviation, and percent change in the motion blur scores of the physician reads for the various DDG_EMDB datasets (100, 50, and 25%) in comparison to the ANZ_EMDB. These results show that while the percent change is relatively large, the differences in the mean motion blurring scores in comparison to the 100% dataset were less than 0.09 indicating that the differences in motion blur are nonappreciable, thereby suggesting that there is no difference in image quality with respect to motion blur as we decrease the percent of the list mode dataset. For the motion blur scores of the three list mode datasets, the inter‐reader intraclass correlation coefficients were 0.69 (moderate), 0.37 (poor), and 0.66 (moderate) for the 100%, 50%, and 25% datasets, respectively. Of the three readers, the highest intra‐rater intraclass correlation coefficients were 1.00 (excellent), 0.78 (good), and 0.59 (moderate) for the 100%, 50%, and 25% list mode datasets, respectively, indicating that the readers ability to repeat their own motion blur scores for 10 patients became more difficult as the amount of data decreased.

**TABLE 1:** Summary of the motion blur scores of the F18‐FDG foci for the for the 100%, 50%, and 25% list‐mode datasets. A value of zero implies that the data driven respiratory gating_elastic motion deblurring (DDG_EMDB) images are similar to the ANZ_EMDB images. Percent change in the mean as well as *p*‐values are calculated with respect to the 100% dataset. The interreader intraclass correlation coefficients (ICC) and the intrareader ICCs are calculated

% Data Used	100%	50%	25%
Mean	0.21	0.3	0.28
Standard deviation	0.43	0.50	0.52
% change		42.86%	33.33%
*p*‐value		0.21	0.21
Inter‐reader ICC	0.69	0.37	0.66
Intra‐reader ICC (R1, R2, R3)	(0.77, 0.79, 1.00)	(0.78, 0.61, 0.73)	(0.00, 0.59, 0.18)

Abbreviation: ICC, intraclass correlation coefficient.

## 4 DISCUSSION

In this paper, we evaluated the impact of decreased injected activity on the performance of a CBM_DDG algorithm with respect to respiratory waveforms, SUV quantification, and physician visual assessment of respiratory motion blur. To the best of our knowledge, this is the first comprehensive evaluation of this CBM_DDG algorithm that has studied the impact of injected activity (count density) on the algorithm. Our investigation showed that as the amount of injected activity was decreased (simulated through removal of PET list mode dataset events), the CBM_DDG waveforms degraded as the percentage data used decreased from 100% to 50% and to 25%, respectively. However, the degradation in waveforms did not have an impact on tumor quantification in the DDG_EMDB images as measurements of SUVmax and SUVpeak showed small changes in comparison to the 100% dataset images. Similarly, the physicians’ evaluation of motion blur in the DDG_EMDB images found that there were negligible differences between the scores of the 100%, 50%, and the 25% list mode datasets.

**FIGURE 5:**
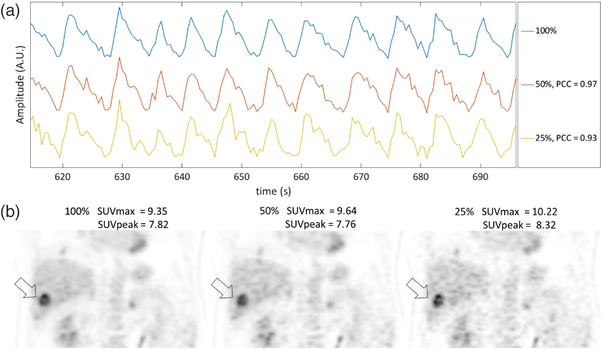
Ideal continuous bed motion_data driven respiratory gating (CBM_DDG) patient with high uptake in liver, kidneys, and tumor with extensive respiratory motion. (a) CBM_DDG respiratory waveforms when using 100%, 50%, and 25% of the list mode data along with respective correlation coefficients. (b) SUVmax of a liver tumor is measured showing that as the amount of data used decreases SUVmax and SUVpeak increases

For the analysis of the patient respiratory waveforms, our finding that the average correlation coefficients decreased as the amount of list mode data decreased is in agreement with prior studies that have demonstrated this result when using different step and shoot DDG algorithms.^30^, ^36^ These waveform results are of concern because using a respiratory waveform that is of poor quality can result in selecting PET data for the motion corrected images that contain unwanted respiratory motion blur. This can ultimately result in underestimated tumor activity quantification and increased respiratory motion blur in the images. In Figure 4, the CBM_DDG waveform for the 25% list mode dataset shows high amounts of noise in the respiratory waveform, and some respiratory peaks and troughs are hard to discern in comparison to those from the 100% and 50% waveforms. For the 25% waveform, it would be expected that the motion‐corrected images would be negatively impacted. However, for some patients the simulated decrease in injected activity had very little impact on the respiratory waveform as seen in Figure 5. The correlation coefficients were very high at 0.97 and 0.93 for the 50% and 25% list mode dataset waveforms, respectively. This patient is an example of the ideal candidate for DDG as the patient had substantial amounts of activity in liver tumors, the liver, and kidneys. In addition, all of these anatomical locations were under the influence of a large amount of respiratory motion making it easier for the CBM_DDG algorithm to determine a respiratory waveform even with decreased count density.

For patients with degraded DDG respiratory waveforms due to decreased injected activity, we expected to see a corresponding decrease in tumor SUVs due to an inferior motion blur correction when using the CBM_DDG waveform. However, this study showed that as the amount of data used decreased, both SUVmax and SUVpeak increased, although by only a small amount for SUVmax and a negligible amount for SUVpeak. This observation can be attributed to an increase in image noise with decreasing amounts of percent data used. Figures 4 and 5 clearly show that the noise in the 50% and 25% list mode data images has greatly increased in comparison to the 100% image. These increases in image noise artifactually increase SUVmax^44^ and could have counteracted any decreases in tumor SUV caused by respiratory motion and utilization of degraded CBM_DDG waveforms. In contrast to SUVmax, there were negligible increases in SUVpeak primarily because this metric averages the SUVs of pixels rather than selecting the maximum pixel value and hence is less susceptible to image noise^44^ suggesting that the degrading CBM_DDG respiratory waveform resulting from decreased injected activity is perhaps counterbalanced by the increased noise with a net effect of negligible impact on tumor quantification.

As the patient waveforms degraded with decreased injected activity, we also anticipated that the physicians would note differences in motion blur when evaluating the DDG_EMDB motion‐corrected images. However, the physician evaluation showed that the differences between the 100%, 50%, and 25% motion blur scores were nonappreciable. In addition, the average motion blur scores of 0.21, 0.3, and 0.28 for the 100%, 50%, and 25% images, respectively, suggest that the degraded DDG waveform had little effect on the visual assessment of the resultant CBM_DDG motion‐corrected images when compared to the ANZ motion‐corrected images. Given that most of the scores occurred between 0 (no blur) and 1(slightly more blur) and to that call differences so small is a difficult task, we expected that the inter‐reader ICCs would decrease as the count density in the images decreased. We believe this is possibly due to the increasing levels of image noise making scoring of the images more difficult. However, the inter‐reader ICC values did not decrease as expected having values of 0.69 (moderate), 0.37 (poor), and 0.66 (moderate) for the 100%, 50%, and 25% datasets, respectively. In addition to the low amounts of motion and increasing levels of noise, this was perhaps caused by the small number of readers that were used in this study. To assess this reliability especially when we have small differences in motion blur, we believe a larger number of readers would be needed. This however is beyond the scope of this work. It is important to note however that the intra‐reader ICC did decrease as the count density decreased from 100% to 25% as we expected. These results suggest that motion blur was not introduced into these images by the degrading CBM_DDG waveforms and that utilization of the CBM_DDG algorithm with decreased injected activity is not likely to degrade the interpretation of these images.

For the physician evaluation of motion blur, we chose not to compare the DDG reconstructions to each other (such as comparing the 100% to the 50% and 25% images) as we did for the waveforms and lesion quantitation since that would likely be biased by the stark differences in resultant image noise alone. Rather, we opted to compare the 100% ANZ_EMDB images to the 100% DDG_EMDB images and likewise for the 50% and 25% list mode data images. Other than the motion blur evaluation, we chose not to include a comparison of the Anzai and DDG waveforms as well as the ANZ_EMDB and DDG_EMDB image quantification has already been thoroughly investigated.^35^ Rather, our focus was to determine the impact of reduced injected activity on the CBM_DDG algorithm.

The investigation in this study focused on evaluating the impact of decreased activity on the performance of the CBM_DDG algorithm and its ensuing results on respiratory waveform, lesion quantification, and physician evaluation of image quality. However, we believe that the results of this work can also be extended to the impact of increasing scan speed on the CBM_DDG algorithm. Scan time and injected activity in CBM PET data acquisition are relatively interchangeable. A decrease in injected activity results in a decrease in the number of detected events similar to that achieved with increasing scan speed.

One limitation of this study was that a free breathing CT was used to perform attenuation correction and could have resulted in inaccuracies in quantification and distortions of the radioactive concentration in the foci when performing attenuation correction due to a mismatch between the PET and the CT. However, given that the same free breathing CT was used for the attenuation correction of all the % DDG images, any bias from such mismatch effects is expected to be the same independent of the % DDG data used. It is important to note however that recently developed methods have shown promise to align the PET data to the CT images.^45^, ^46^

An additional limitation of this work is that the list mode datasets with simulated lower injected activity have different noise profiles in comparison to scans acquired with lower injected activity. We acquired our patient scans with 320.7 ± 54.4 MBq ^18^F‐FDG, which will have a higher randoms fraction, in comparison to a scan with 50% and 25% of this injected activity. We created simulated decreased injected activity datasets by randomly removing a fraction of the coincidence events in the original list mode dataset through decimation. Unfortunately, this preserves the higher randoms fraction creating concern that the reconstructed image noise and quantification will be different than a scan with lower injected activity. However, a recent publication^47^ has shown that these differences in the noise profiles when using this decimation tool to create simulated lower injected activity datasets have negligible impact on image noise and quantification. To investigate this, the paper performed an in‐human study that compared low injected activity PET scans to simulated low injected activity scans, which were created from a high injected activity scan.

Another limitation of this work is our lack of evaluating the DDG algorithm in a more controlled environment such as a phantom study where we could have physically reduced the amount of activity in the phantom. This was out of the scope of this work because it would necessitate the construction of a specialized phantom that has multiple compartments of various activity concentrations that move with respect to one another to mimic the anterior‐posterior motion of the abdominothoracic cavity to allow the successful implementation of the CBM_DDG algorithm. Such a phantom could also support investigating the impact of lesion size, lesion contrast, and lesion motion on the CBM_DDG algorithm.

In this work, only patients scanned with F18‐FDG were included in the research protocol, and future work should investigate the performance of the CBM_DDG algorithm with radiotracers that are gaining more widespread use clinically such as Ga‐68 DOTATATE for neuroendocrine studies and F18‐Fluciclovine or PSMA labeled agents for prostate cancer. The differing biodistributions of these and other tracers in normal and diseased tissue might be beneficial or pose a challenge to the CBM_DDG algorithm.

Finally, as future PET scanner technology improves, so too should the CBM_DDG algorithm. The recently introduced Siemens Biograph Vision,^48^ which is capable of CBM, has a sensitivity that is at least 70.3% higher than the mCT Flow used in this study and should improve the quality of the CBM_DDG signal, although it is likely that the higher sensitivity will be traded in to reduce the total injected activity. In addition, the time‐of‐flight resolution of the Biograph Vision system has improved substantially from 540 ps to 210 ps, which should also improve the signal to noise ratio of the time of flight histo‐projections, which are central to the CBM_DDG algorithm (Figure 1).

## 5 CONCLUSION

This work shows that although the CBM_DDG waveform degrades in quality with decreasing count density, the resultant respiratory motion‐corrected images have negligible effect on lesion quantification and image quality.

## AUTHOR CONTRIBUTIONS

Dr. Joseph Meier participated in the study design, identified candidate patients, oversaw patient examinations, commissioned research software, reconstructed and analyzed images, organized physician reads, and analyzed all data and was the primary composer of the manuscript. Dr. Radwan Diab and Trevor Connor contributed to the composition and editing of the manuscript. Dr. Osama Mawlawi is the principal investigator of this research project and has provided extensive input on the design of the experiments and editing of the manuscript.

## FUNDING INFORMATION

Siemens Medical Solutions USA, Inc.

## CONFLICT OF INTEREST

The authors declare that there is no conflict of interest that could be perceived as prejudicing the impartiality of the research reported.
